# Low-Temperature One-Pot Fabrication of a Dual-Ion Conductive Hydrogel for Biological Monitoring

**DOI:** 10.3390/s26072086

**Published:** 2026-03-27

**Authors:** Xinyu Guan, Xudong Ma, Ruixi Gao, Qiuju Zheng, Changlong Sun, Yahui Chen, Jincheng Mu

**Affiliations:** 1 College of Chemistry and Chemical Engineering, Tarim University, Alar 843300, China; 15516004509@163.com (X.G.); 18196871108@163.com (X.M.); gaoruiqian8888@163.com (R.G.); happysunchanglong@126.com (C.S.); 2 Engineering Laboratory of Chemical Resources Utilization in South Xinjiang, Xinjiang Production & Construction Corps, Alar 843300, China; 3 School of Materials Science and Engineering, Qilu University of Technology, Jinan 250353, China; qlzhengqj@163.com

**Keywords:** conductive hydrogel, dual-ion conduction, low-temperature polymerization, anti-freezing hydrogel, wearable biosensors

## Abstract

Flexible conductive hydrogels hold great promise in wearable electronics and biomonitoring applications, yet their practical use is constrained by issues such as poor low-temperature tolerance, susceptibility to dehydration, and limited multifunctional sensing capabilities. This study successfully synthesized a dual-conductive lithium-ion and calcium-ion hydrogel based on acrylamide/gelatin via a simplified low-temperature one-pot polymerization method. At 60 °C, mixing acrylamide, gelatin, lithium chloride, and calcium chloride within 40 min constructed a network structure featuring covalent bonds, ionic bonds, and hydrogen bonds. The resulting material exhibited exceptional extensibility with a break elongation of 1408.5% and tensile strength of 134.2 kPa while maintaining strong adhesion to nine different substrates. It retained flexibility at −20 °C and demonstrated minimal mass loss (3% of initial value) after 10 days of aging. As a sensor, this hydrogel reliably responds to pressure, temperature, large-amplitude body movements, and subtle physiological signals like pulse and vocal cord vibrations. In animal simulation monitoring, its electrical resistance signals increased linearly with model body weight and remained stable between −20 °C and 20 °C. These results demonstrate the hydrogel’s broad application potential in wearable sensing, ecological monitoring, and smart agriculture.

## 1. Introduction

The rapid advancement of wearable electronics, human–machine interaction systems, and personalized health monitoring technologies has created an urgent demand for flexible conductive materials that combine mechanical adaptability, environmental stability, and high sensing performance [1,2,3,4]. Traditional conductive materials, such as metals, semiconductors, and rigid polymers, typically suffer from poor flexibility, limited biocompatibility, and mismatched mechanical properties with biological tissues, severely limiting their application in scenarios requiring close contact with human skin or animal surfaces [5,6,7]. Hydrogels, as soft materials featuring multidimensional crosslinked networks, have emerged as strong candidates for flexible conductive platforms due to their inherent hydrophilicity, tunable mechanical properties, and excellent biocompatibility [8,9,10,11,12,13,14,15,16,17,18,19,20,21,22,23,24]. However, conventional hydrogels face critical bottlenecks hindering their practical application: cumbersome preparation processes (e.g., prolonged high-temperature polymerization or UV irradiation) with high energy consumption, susceptibility to freezing at subzero temperatures (leading to structural collapse and loss of conductivity), and prone to dehydration under ambient conditions (resulting in mechanical brittleness and functional failure). These limitations have become major obstacles to the development of next-generation flexible conductive materials.

To address the mechanical brittleness of hydrogels, researchers have explored multi-network design strategies. For instance, Zhao et al. developed a sodium alginate/polyacrylamide (SA/PAM) semi-interpenetrating network hydrogel that achieved a tensile strength of 266 kPa and a self-healing efficiency of 99% through dynamic hydrogen bonding [25,26,27,28,29,30]. Ma et al. prepared a polyacrylamide-gelatin-MXene composite hydrogel exhibiting over 1000% tensile strain and a strain coefficient (GF) of 1.10 [31,32,33,34]. While these designs enhance mechanical properties, they often fail to simultaneously address environmental stability issues (e.g., freeze resistance and dehydration resistance) and remain reliant on energy-intensive preparation methods. Furthermore, integrating high conductivity, strong adhesion to diverse substrates, and multi-stimulus responsiveness (e.g., strain, pressure, temperature) within a single hydrogel system remains a significant challenge. Ionic conductive hydrogels, which utilize mobile ions for electrical conduction, have garnered attention due to their simple preparation, high conductivity, and excellent biocompatibility [34,35,36]. Inorganic salts (e.g., LiCl, CaCl_2_) are commonly introduced to provide free ions, though their role is often limited to enhancing conductivity. Recent studies indicate that certain salts can also modulate the hydrogen-bond networks of water molecules to improve freeze resistance. For instance, Cui et al. prepared a PAM/gelatin/SA-Ca^2+^/LiCl hydrogel that maintained flexibility at −20 °C by disrupting ice crystal formation using Ca^2+^ [37]. However, the dehydration resistance of these hydrogels remains inadequately addressed, and their adhesion to non-polar substrates (e.g., PTFE, plastics) remains poor, limiting their application on complex surfaces.

Biomass-derived materials such as gelatin offer unique advantages in hydrogel preparation due to their excellent biocompatibility, inherent adhesiveness, and temperature responsiveness [38,39]. Gelatin, derived from collagen hydrolysis, contains abundant amino, hydroxyl, and carboxyl groups that form hydrogen bonds with synthetic polymers like PAM, enhancing mechanical flexibility and adhesion. Yan et al. demonstrated that gelatin/PAM dual-network hydrogels exhibit high tensile strength (268 kPa) and biocompatibility, yet their self-healing efficiency at physiological temperatures was only 53%, with limited environmental stability [26]. Therefore, combining biomass-derived materials with functional inorganic salts offers a promising strategy for designing hydrogels with balanced mechanical properties, adhesion, and environmental stability.

This paper reports a novel multifunctional ion-conductive hydrogel (denoted as AG/Li^+^/Ca^2+^) prepared via a simple, low-energy-consumption method using acrylamide (AM), gelatin, lithium chloride (LiCl), and calcium chloride (CaCl_2_). This hydrogel rapidly gels within 40 min at 60 °C, eliminating the need for prolonged high-temperature processing or UV irradiation. The synergistic effect of Li^+^ and Ca^2+^ plays a crucial role in enhancing the hydrogel’s comprehensive properties: Li^+^ provides abundant free ions for high conductivity, while Ca^2+^ forms dynamic ionic crosslinks with gelatin and PAM chains to strengthen the network structure and improve freeze/dehydration resistance. Gelatin not only enhances biocompatibility but also contributes strong adhesion to diverse substrates (e.g., wood, pig skin, metals, plastics) through multi-site molecular interactions (hydrogen bonds, electrostatic interactions). This work addresses key limitations of conventional hydrogels by integrating simple preparation, mechanical robustness, environmental stability, and multifunctional sensing into a single system. The proposed AG/Li^+^/Ca^2+^ hydrogel not only provides a novel material platform for flexible conductive applications but also offers a promising strategy for designing next-generation wearable electronics, personalized medical devices, and precision animal monitoring systems.

## 2. Experiment

### 2.1. Materials

Gelatin (Type A, derived from pig skin) was supplied by Shanghai Aladdin Bio-Chem Technology Co., Ltd. (Shanghai, China). Acrylamide (AM) was purchased from Shanghai Aladdin Bio-Chem Technology Co., Ltd. (Shanghai, China). N, N′-Methylenebisacrylamide (MBA) was purchased from Tianjin Damiao Chemical Reagent Factory. (Tianjin, China), ammonium persulfate (APS) from Tianjin Beilian Fine Chemical Development Co., Ltd. (Tianjin, China), lithium chloride (LiCl) from Shanghai Shanpu Chemical Co., Ltd. (Shanghai, China). and anhydrous calcium chloride (CaCl_2_) from Tianjin Zhiyuan Chemical Reagent Co., Ltd. (Tianjin, China). All chemicals were of analytical grade and used as received without further purification.

### 2.2. Preparation of Hydrogel

Preparation of AM/gelatin organic hydrogels (AG/Li^+^/Ca^2+^) via a one-pot method. First, gelatin was dissolved in an appropriate amount of water at 60 °C in a constant-temperature water bath. Acrylamide (AM) was then added to achieve a final concentration of 2.01 mol/L, along with N,N′-methylenebisacrylamide (MBA) as a crosslinking agent at 0.5% molar ratio relative to AM. The mixture was stirred continuously at room temperature for 5 min to promote dissolution. Subsequently, LiCl and CaCl_2_ were added sequentially. The solution was purged with high-purity nitrogen for 5 min to remove dissolved oxygen. The solution underwent 5 min of ultrasonic treatment to eliminate bubbles. Ammonium persulfate (APS) at 0.2% molar concentration relative to AM was then added as an initiator and stirred until completely dissolved. The resulting solution was poured into a mold and reacted at 60 °C for 40 min. After the reaction, the formed hydrogel was removed from the mold, yielding the AG/Li^+^/Ca^2+^ hydrogel composed of AM, gelatin, LiCl, and CaCl_2_.

### 2.3. Characterization

The morphology of the composite hydrogel was observed using scanning electron microscopy (SEM) (Hitachi S4800). Samples were freeze-dried to completely remove water from the hydrogel network. Subsequently, freeze-dried sections were sputter-coated with gold and examined by SEM at an acceleration voltage of 6 kV. The Fourier transform infrared (FTIR) spectra of the samples were acquired using a VQF-530A Fourier transform infrared spectrometer (AC 220 V ± 22 V, 50 Hz, 18% humidity, Beijing Beifeng Rayleigh Analytical Instrument (Group) Co., Ltd.), covering a frequency range of 400 to 4000 cm^−1^.

### 2.4. Mechanical Performance Test

Using the electric tensile testing machine (multi-functional version) from Dongguan Zhique Precision Instrument Co., Ltd., uniaxial tensile tests were conducted at room temperature. The tensile speed was set to 35 mm/min. Hydrogel samples were prepared into rectangular specimens (24 mm × 4 mm × 2.5 mm). Fracture work was estimated from the stress–strain curve. Dissipated energy was calculated as the area under the loading–unloading curve.

### 2.5. Adhesion Test

The interfacial adhesion properties of the hydrogels were systematically tested, with a focus on evaluating their bonding strength on various substrates, including wood, pig skin, copper, aluminum, polytetrafluoroethylene (PTFE), steel, glass, plastic, and ceramics. In the experiments, hydrogel samples were cut into 30 mm × 30 mm squares and bonded between two identical substrates to form a symmetrical adhesive structure. Bonding performance was evaluated using tensile shear tests conducted on an electric tensile testing machine at a strain rate of 5 mm·min^−1^. The corresponding bond strength was calculated by recording the maximum force achieved during loading and combining it with the actual contact area of the bonded interface.

### 2.6. Environmental Resistance Testing

To investigate the moisture retention properties of the hydrogels, samples were placed in a constant temperature and humidity environment maintained at approximately 30 °C and 80% relative humidity. Their mass changes were measured periodically, and the moisture retention rate or weight change rate of the hydrogels was calculated using the following formula: ΔW = (W_0_ − W_t_)/W_0_, Δ*W* represents the percentage change in mass of the hydrogel, where W_0_ and W_t_ denote the initial mass and mass at time t of the hydrogel, respectively.

### 2.7. Electrical Signal Testing

Using a Keithley 2400 digital multimeter, we measured the electrical signal changes in conductive hydrogels during human movement. To detect physiological activities such as finger flexion, wrist flexion, swallowing, and heartbeat pulses, hydrogel samples were cut into circular pieces (radius = 5 mm, thickness = 1 mm) and encapsulated with PDMS to prevent moisture evaporation while enhancing adhesion to corresponding body areas. Two copper foils were connected by soldered copper wires and clamped to opposite ends of the circular hydrogel. The other ends of the copper wires were connected to the positive and negative terminals of the Keithley 2400, respectively. Under a constant voltage of 30 V, the Keithley 2400 monitored the relative resistance change of the hydrogel sensor, calculated by the following equation: ΔR/R_0_ = (R − R_0_)/R_0_, where R_0_ is the initial resistance and R is the resistance after deformation.

## 3. Results and Discussion

### 3.1. Synthesis and Characterizations of AG/Li^+^/Ca^2+^ Hydrogels

**Figure 1:**
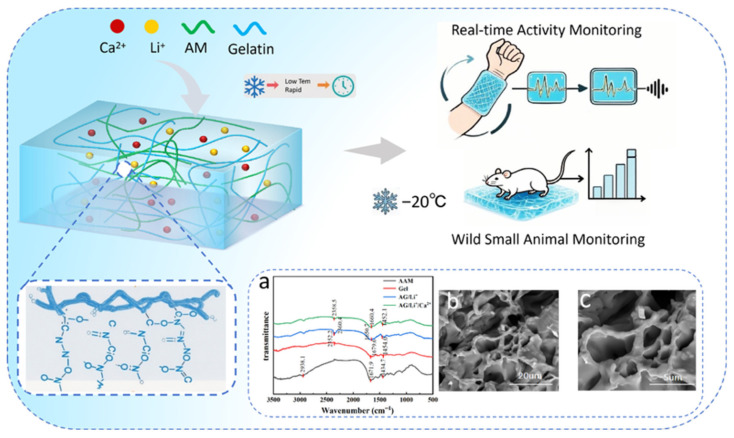
Schematic diagram of AG/Li^+^/Ca^2+^ hydrogel synthesis mechanism and diversified applications. (**a**) Spectra of AG/Li^+^/Ca^2+^ hydrogel. (**b**,**c**) SEM image of AG/Li^+^/Ca^2+^ hydrogel.

Figure 1 presents the schematic diagram of the synthesis mechanism for the AG/Li^+^/Ca^2+^ hydrogel, its Fourier transform infrared (FTIR) spectrum (Figure 1a), and scanning electron microscopy (SEM) image (Figure 1b,c). Figure 1 clearly illustrates the process of preparing the hydrogel via a one-pot radical polymerization method at 60 °C for 40 min, using AM and gelatin (Gel) as the matrix, LiCl and CaCl_2_ as functional additives. Li^+^ ions were incorporated through electrostatic interactions with the polymer’s polar groups, while Ca^2+^ ions formed a complex with the polymer chains and CaCl_2_ as functional additives. Li^+^ ions bind to polymer polar groups via electrostatic interactions to provide mobile ions, while Ca^2+^ ions form a dynamic crosslinking network through coordination bonds. This is consistent with the formation of a dual-ion-regulated network involving covalent crosslinks, hydrogen-bonding interactions, and dynamic ionic associations. Figure 1a shows the FTIR spectra comparing pure AM (black line), pure gel (red line), AG complex (blue line), and AG/Li^+^/Ca^2+^ hydrogel (green line). Pure AM exhibited characteristic peaks at 2938.1 cm^−1^ (C-H stretching) and 1671.9 cm^−1^ (C=O stretching), pure gel exhibited characteristic peaks at 1679 cm^−1^ (amide I band) and 2352.2 cm^−1^ (amide II band). The AG complex spectrum overlaps these features with peak shifts (e.g., 1671.9 cm^−1^ → 1665 cm^−1^). The AG/Li^+^/Ca^2+^ spectrum exhibited new absorption bands at 2360 cm^−1^ and 1650 cm^−1^ (Li^+^ effect), with complex peak shapes in the low-wavenumber region (Ca^2+^ coordination effect), consistent with the formation of a dual-ion-regulated network. The cross-sectional SEM images (Figure 1b,c and Figure S1) show the porous structure of the hydrogel with uniform pore sizes (approximately 5–20 μm). This structure provides unobstructed pathways for ion migration while ensuring mechanical stability, establishing the structural foundation for its application as a flexible sensor.

### 3.2. Mechanical Properties of AG/Li^+^/Ca^2+^ Hydrogels

**Figure 2:**
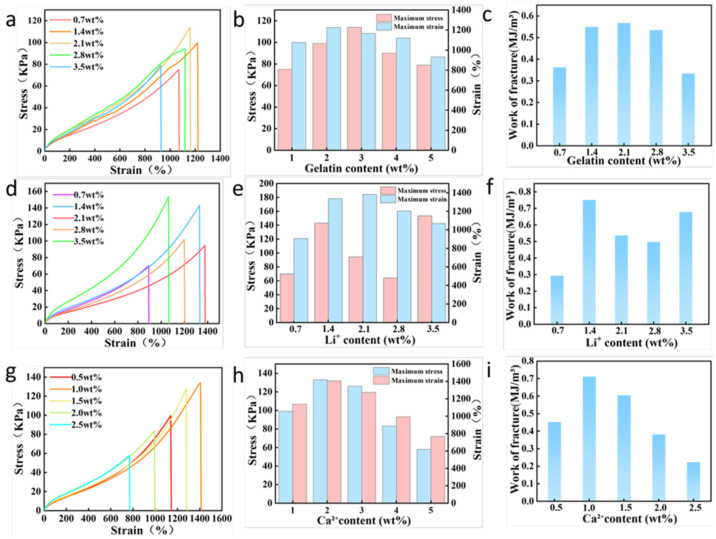
(**a**–**c**) Tensile curves, three-dimensional tensile curves, and fracture work of hydrogels with different gelatin contents. (**d**–**f**) Tensile curves, three-dimensional tensile curves, and fracture work of hydrogels with different lithium ion contents. (**g**–**i**) Tensile curves, three-dimensional tensile curves, and fracture work of hydrogels with different calcium ion contents.

Figure 2 presents the tensile mechanical properties and cyclic stability test results of AG/Li^+^/Ca^2+^ hydrogels at different component concentrations. As shown in Figure 2a–c, Table S1, the existence of gelatin significantly modulates the mechanical properties of hydrogel, with fracture stress, fracture strain, and fracture work exhibiting a “first increase, then decrease” trend as gelatin content rises. When the gelatin concentration was 2.1 wt%, the hydrogel exhibited optimal mechanical properties, achieving a maximum stress of 114.4 kPa, a corresponding fracture strain of 1160.3%, and a high fracture energy of 0.56695 MJ/m^3^. This is attributed to the unique triple-helix structure of gelatin molecules, which significantly enhances hydrogel toughness through energy dissipation effects. However, excessively high gelatin content leads to the over-packing of molecular chains, weakening the entanglement within the polymer network, and causing a decline in mechanical properties. Consequently, all subsequent experiments employed 2.1 wt% as the optimal gelatin content. Figure 2d–f and Table S2, illustrates the the influence of Li^+^ concentration on hydrogel mechanical properties, exhibiting the similar trend with the gelatin. When the Li^+^ concentration reached 1.4 wt%, the hydrogel showed the uppermost mechanical properties, and the corresponding maximum stress was 143.2 kPa, the fracture strain was 1330.0%, and the fracture energy was 0.7505 MJ/m^3^. From a mechanism perspective, the appropriate Li^+^ concentration can optimize the hydrogel mechanical properties by disrupting hydrogen bonding between polymer chains and reducing excessive entanglement within the network. However, excessively high Li^+^ content compromises polymer network integrity, leading to simultaneous decreases in both tensile stress and strain at break.

The effect of calcium ion (Ca^2+^) content on hydrogel tensile properties is shown in Figure 2g–i and Table S3. As Ca^2+^ concentration increases, the ionic crosslinking between Ca^2+^ and polar groups on polymer chains gradually intensifies, enhancing network crosslink density and simultaneously improving both the tensile stress and strain of the hydrogel. When Ca^2+^ concentration reached 1.0 wt%, the hydrogel exhibited optimal mechanical properties with a maximum stress of 134.2 kPa, a fracture strain of 1408.5%, and a fracture work of 0.71195 MJ/m^3^. Further increasing Ca^2+^ concentration led to excessive ionic crosslinking, causing densification of the polymer network structure and a significant decrease in toughness, ultimately degrading the hydrogel’s tensile properties. Therefore, 1.0 wt% was selected as the optimal Ca^2+^ concentration for subsequent experiments. Figure S2a,b shows the cyclic tensile stability test results for the AG/Li^+^/Ca^2+^ hydrogel. Figure S2a shows that within the 100–1200% strain range, the hysteresis loop morphology of the hydrogel loading–unloading curve remains essentially stable. Each loading curve highly overlaps with the preceding unloading curve, indicating that physical bonds (e.g., hydrogen bonds, ionic bonds) dissociated during loading can rapidly re-form upon unloading. Figure S2b demonstrates that after five cyclic stretches at a constant strain of 700%, the hysteresis loops remained largely superimposed, with no significant decay in dissipated energy. These results confirm that the AG/Li^+^/Ca^2+^ hydrogel exhibits outstanding tensile stability and rapid energy dissipation recovery capability.

### 3.3. Adhesion Properties of AG/Li^+^/Ca^2+^ Hydrogels

**Figure 3:**
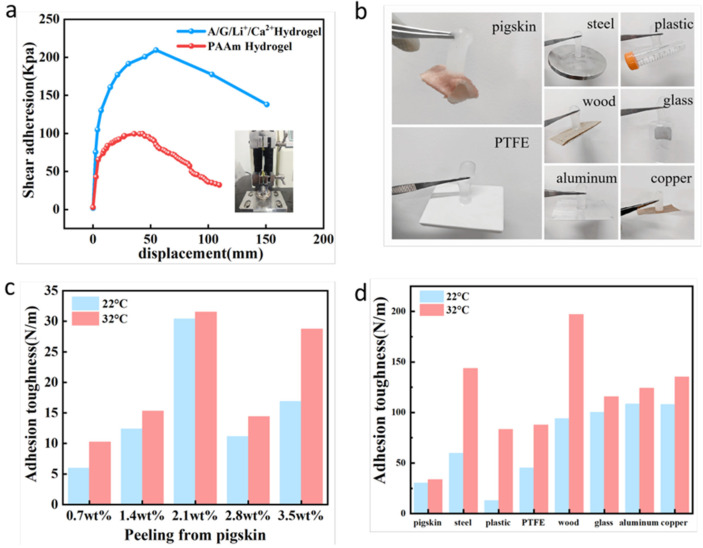
(**a**) Photograph showing the adhesion strength and peel test of hydrogel to glass at 180° measured using an electric tensile testing machine. (**b**) Demonstration of hydrogel adhesion to different solid material surfaces. (**c**) Adhesion strength of hydrogels with varying gelatin contents to pig skin at different temperatures. (**d**) Adhesion strength of hydrogels with identical gelatin content to different solid materials at varying temperatures.

Figure 3a and Figure S3 illustrates the schematic and results of the 180° peel test between the hydrogel and glass substrate. A 50 μm biaxially oriented polypropylene film was used as the rigid backing to restrict elongation during hydrogel peeling, with a peel speed set at 50 mm/min. Results indicate that the AG/Li^+^/Ca^2+^ hydrogel exhibits a maximum adhesion force of 151 N/m to glass, significantly higher than the 70.1 N/m observed for the pure polyacrylamide (PAM) hydrogel. This difference stems from the active functional groups in gelatin molecules—including amino, hydroxyl, and carboxyl groups—forming strong hydrogen bonds with hydroxyl groups on the glass surface. Simultaneously, the dissociation of gelatin’s triple-helix structure at the test temperature enhances its fluidity, promoting close contact between the hydrogel and the glass surface, thereby further strengthening adhesion. Figure 3b visually demonstrates the adhesion capability of the AG/Li^+^/Ca^2+^ hydrogel on nine substrate types: wood, pig skin, copper, aluminum, polytetrafluoroethylene (PTFE), steel, glass, plastic, and ceramic. After heating, the hydrogel stably adhered to various substrates without significant detachment, demonstrating broad substrate adaptability. For polar substrates (e.g., glass, metals), adhesion primarily relies on hydrogen bonds and ionic interactions; for nonpolar substrates (e.g., PTFE, plastics), it is achieved through molecular chain penetration and van der Waals forces. This reflects the universal adhesion properties of gelatin synergistically combined with PAM.

Figure 3c compares the adhesion strength of hydrogels with different gelatin contents to pig skin at 22 °C and 32 °C. Results show that gelatin content significantly regulates adhesion performance: when gelatin concentration reached 2.1 wt%, the hydrogel exhibited peak adhesion strength to pig skin, and the strength at 32 °C consistently exceeded that at 22 °C. This occurs because 2.1 wt% gelatin forms an optimal density of intermolecular interaction sites (hydrogen bonds and electrostatic interactions with porcine collagen), while elevated temperature promotes gelatin chain motion, enhancing contact compatibility with the porcine skin surface. Conversely, excessively high gelatin content leads to excessive molecular chain aggregation, weakening interfacial interactions and reducing adhesion strength. Figure 3d compares the adhesion strength of the hydrogel at 22 °C and 32 °C to different substrates at a 2.1 wt% gelatin content. At 32 °C, the hydrogel exhibited higher adhesion strength than at 22 °C on all tested substrates, with particularly significant increases (20–35%) on pig skin, steel, and aluminum substrates. Mechanistically, 32 °C approached gelatin’s gel–sol transition temperature range, enhancing molecular chain mobility and enabling better adaptation to substrate surface topography. Simultaneously, elevated temperature accelerates the interaction kinetics between gelatin and surface functional groups of the substrate, further strengthening interfacial bonding. It should be noted that excessively high temperatures (e.g., above 40 °C) may cause gelatin degradation or excessive hydrogel swelling, thereby reducing adhesion stability.

### 3.4. Freeze Resistance and Environmental Stability of AG/Li^+^/Ca^2+^ Hydrogels

**Figure 4:**
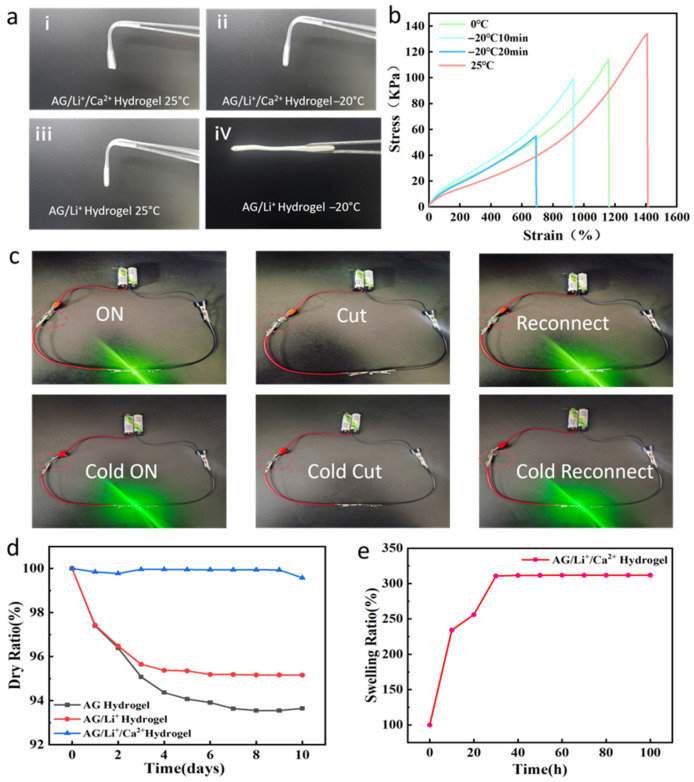
(**a**) Comparison of AG/Li^+^/Ca^2+^ and AG/Li^+^ hydrogels before and after 20 min at −20 °C. (**b**) Tensile stress–strain curves of AG/Li^+^/Ca^2+^ hydrogels at different temperatures. (**c**) AG/Li^+^/Ca^2+^ hydrogel at room temperature: cutting it still allowed for powering a small light bulb; AG/Li^+^/Ca^2+^ hydrogel reconnected after cutting at −20 °C still powered a small light bulb. (**d**) Relative change curves over time for AG hydrogel, AG/Li^+^ hydrogel, and AG/Li^+^/Ca^2+^ hydrogel at room temperature. (**e**) Expansion rate experiment for the AG/Li^+^/Ca^2+^ hydrogel.

Conventional hydrogels are prone to freezing in low-temperature environments or dehydrate and harden at room temperature due to water evaporation. This severely limits their practical application as flexible conductive materials. This study introduced CaCl_2_ to impart excellent freeze resistance and dehydration resistance to the hydrogel. Figure 4a compares the macroscopic states of the AG/Li^+^/Ca^2+^ and AG/Li^+^ hydrogels after 20 min at −20 °C. The AG/Li^+^ hydrogel exhibited noticeable freezing and hardening, while the AG/Li^+^/Ca^2+^ hydrogel remained soft and deformable, indicating that Ca^2+^ effectively suppresses ice crystal formation. The mechanism involves CaCl_2_ acting as a strong electrolyte: upon dissolution, released Ca^2+^ and Cl^−^ ions disrupt the hydrogen bond network between water molecules, weaken their ordered aggregation, and significantly lower the freezing point of water within the hydrogel. Additionally, the solvation layer formed by ionic hydration inhibits ice nucleation and growth [37]. Figure 4b shows the tensile stress–strain curves of the AG/Li^+^/Ca^2+^ hydrogel at 25 °C, 0 °C, and −20 °C (10 min, 20 min). Results indicate that although the gel’s mechanical properties declined with decreasing temperature, it maintained a tensile strength of 54.8 kPa and elongation at break of 691.1% after 20 min at −20 °C, significantly outperforming the AG/Li^+^ gel (which exhibited near-complete brittle fracture at −20 °C with strain < 50%). This result confirms that Ca^2+^ incorporation not only confers freeze resistance to the hydrogel but also preserves its flexibility and tensile toughness at low temperatures, meeting the requirements for use in cryogenic environments.

To verify the conductive stability of the AG/Li^+^/Ca^2+^ hydrogel under extreme conditions, a circuit on–off test was designed as shown in Figure 4c. The hydrogel was integrated into a closed circuit and connected to an indicator light to examine its conductive stability at both room temperature and −20 °C. Experimental results demonstrate that regardless of ambient or −20 °C conditions, the hydrogel restored circuit conductivity upon reconnection after disconnection, with the indicator light functioning normally. This phenomenon not only confirms that the ion-conductive hydrogel maintains ion migration capability and dynamic coordination bonds with low-temperature self-healing properties at low temperatures but also indicates the temperature adaptability of the AG/Li^+^/Ca^2+^ conductive hydrogel system. This phenomenon may be attributed to CaCl_2_ acting as a strong electrolyte. The dissociated Ca^2+^ and Cl^−^ ions not only provide charge carriers but also suppress ice crystal formation by reducing water activity. The amino and carboxyl groups abundant in gelatin molecular chains form reversible coordination bonds with Ca^2+^. These bonds can rapidly reconnect after temporary disruption under external force. This dynamic cross-linked network endows the material with low-temperature self-healing capability and morphological stability. Experimental results demonstrate that the AG/Li^+^/Ca^2+^ hydrogel successfully achieved synergistic enhancement of freeze resistance, conductivity, and self-healing through an ion-regulation strategy, providing a material foundation for developing novel flexible electronic devices suitable for extreme environments.

To systematically evaluate the environmental stability of the AG/Li^+^/Ca^2+^ hydrogel, this study comprehensively examined its water retention capacity, moisture absorption characteristics, and swelling behavior. By comparing the mass changes of AG, AG/Li^+^, and AG/Li^+^/Ca^2+^ hydrogels after 10 days of storage at 25 °C and 80% relative humidity, their dehydration resistance was systematically assessed. As shown in Figures S4 and Figure 4d, the AG/Li^+^/Ca^2+^ hydrogel demonstrated outstanding water retention, maintaining 97% of its initial mass after 10 days—significantly outperforming the control group. Quantitative analysis in Figure 4d further revealed that its mass loss rate was approximately 3.5 times lower than that of the AG hydrogel. This outstanding water retention stems from multifaceted mechanisms of Ca^2+^: first, Ca^2+^ forms a stable hydration layer through strong coordination with water molecules, effectively reducing the system’s vapor pressure; second, the hygroscopic nature of Cl^−^ ions enables the material to capture moisture from the environment, establishing a dynamic water balance; Finally, Ca^2+^ cross-links with the polymer network, enhancing network stability and inhibiting the formation of water evaporation pathways. To further evaluate the hydrogel’s water resistance, its swelling behavior in distilled water (pH ≈ 7) was systematically studied. As shown in Figure 4e, the AG/Li^+^/Ca^2+^ hydrogel reached swelling equilibrium within 30 h, with an equilibrium swelling rate of approximately 300%. Notably, even after prolonged immersion for 100 h, the hydrogel maintained complete structural integrity without rupture or dissolution, demonstrating exceptional stability of its crosslinked network. This stability is attributed to the dynamic ionic crosslinking network formed between Ca^2+^ ions and polymer chains (amino and carboxyl groups of gelatin, and amide groups of PAM). On the one hand, ionic crosslinking suppresses excessive swelling of polymer chains; on the other hand, hydrogen bonding interactions between gelatin and PAM further enhance network density, preventing structural damage caused by excessive water penetration. This property ensures the functional stability of the hydrogel in humid environments or brief water exposure (e.g., sweat contact, minor water stains), supporting practical wearable applications for flexible sensors.

### 3.5. AG/Li^+^/Ca^2+^ Hydrogel Pressure-Strain Sensing and Temperature Sensing

**Figure 5:**
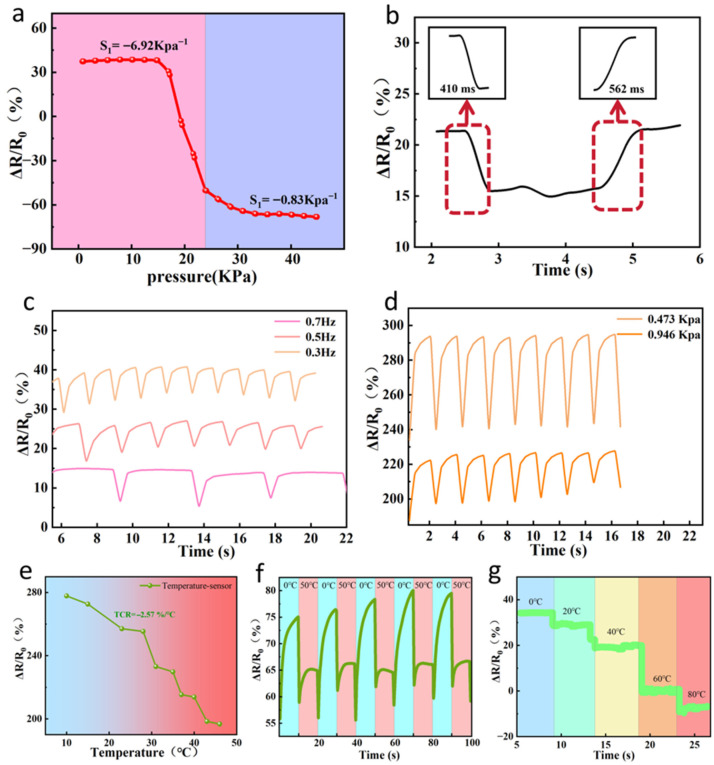
(**a**) Pressure sensitivity of the AG/Li^+^/Ca^2+^ hydrogel sensor. (**b**) Response time and relaxation time of the AG/Li^+^/Ca^2+^ hydrogel sensor at 0.5 kPa. (**c**) Relative resistance change of the AG/Li^+^/Ca^2+^ hydrogel sensor at different frequencies under 0.5 kPa pressure. (**d**) Relative resistance change of the AG/Li^+^/Ca^2+^ hydrogel sensor at different pressures under 0.5 Hz frequency. (**e**) Relative resistance change of the A/G/Li^+^/Ca^2+^ hydrogel sensor as a temperature sensor within the 10–50 °C range. (**f**) Resistive response of A/G/Li^+^/Ca^2+^ hydrogel sensor during cyclic temperature testing between 5 °C and 50 °C. (**g**) Transient thermal response of A/G/Li^+^/Ca^2+^ hydrogel sensor to different temperatures.

Sensitivity is a crucial parameter for evaluating pressure sensor performance, reflecting the relationship between applied pressure and corresponding resistance change. As shown by the slope of the sensitivity definition curve for the hydrogel in Figure 5a, the value ranged from −6.92%/kPa within 0 to 24 kPa, indicating significant changes in the conductive network and demonstrating the hydrogel’s excellent linear response capability. Furthermore, when the hydrogel pressure exceeded 24 kPa, the measured value dropped to −0.83%/kPa, confirming the highly sensitive response characteristics within the hydrogel. Beyond sensitivity, response time and relaxation time are also critical indicators for evaluating pressure sensor performance. In Figure 5b, under a 0.5 kPa applied pressure, the hydrogel exhibited a response time of only 0.41 s and a relaxation time of just 0.56 s, demonstrating rapid electrical signal response and recovery capability. To further validate the hydrogel’s stability under varying frequencies at constant pressure, sensor response signals were measured at different frequencies while maintaining a fixed pressure. As shown in Figure 5c, the resistance curves at different frequencies almost perfectly overlapped, demonstrating excellent frequency adaptability and stability. Additionally, under the same frequency but different pressure conditions (Figure 5d), the sensor electrical signal response of the tested hydrogel indicates that the measured signal amplitude increases correspondingly with increasing pressure, exhibiting good pressure recognition capability and response stability. Pressure strain sensing tests revealed that the pressure sensor in this hydrogel not only exhibits high sensitivity and a wide response range but also demonstrates fast response and recovery times, along with good recognition capabilities under various frequency and pressure conditions.

This hydrogel not only exhibits outstanding pressure-responsive properties but also demonstrates excellent thermal sensitivity. This opens up significant possibilities in the field of temperature sensing. To quantify the sensitivity of its thermal response, we introduced the temperature coefficient of resistance (TCR). This parameter is defined as the relative change in resistance value of a material with temperature variation, typically expressed as: TCR = (1/R_0_) × (ΔR/ΔT), where R_0_ is the initial resistance, ΔR represents the rate of resistance change with temperature, and ΔT is the temperature change. This parameter effectively reflects the resistance variation trends of hydrogels under different environmental conditions and serves as a crucial basis for evaluating their temperature sensing capabilities. As shown in Figure 5e, this hydrogel-based temperature sensor exhibits a distinct negative temperature coefficient (NTC) behavior, with resistance decreasing as temperature increases. During heating and cooling cycles, the hydrogel demonstrates highly linear thermal response characteristics, indicating excellent reversibility and stability. Its resistance temperature coefficient (TCR) of −2.57%/°C indicates the sensor’s capability for rapid and sensitive electrical signal response to temperature changes, enabling real-time temperature monitoring and feedback. Figure 5f shows testing at different temperatures. When the ambient temperature rises to 50 °C, the resistance decreases rapidly due to its temperature characteristics, while it increases rapidly under cooling conditions. After five cycles between 0–50 °C, the resistance response remains consistently accurate, demonstrating high-performance sensing capability. To further validate the thermal response capability of the hydrogel-based sensor in practical applications, dynamic temperature monitoring was conducted. As shown in Figure 5g, the resistance of the hydrogel exhibited a stepwise decrease during cooling, subsequently reaching a new equilibrium with the surrounding environment. These results fully demonstrate the hydrogel’s outstanding thermal response speed and excellent repeatability, making it suitable for temperature monitoring.

### 3.6. Electro-Mechanical Properties of AG/Li^+^/Ca^2+^ Hydrogels

**Figure 6:**
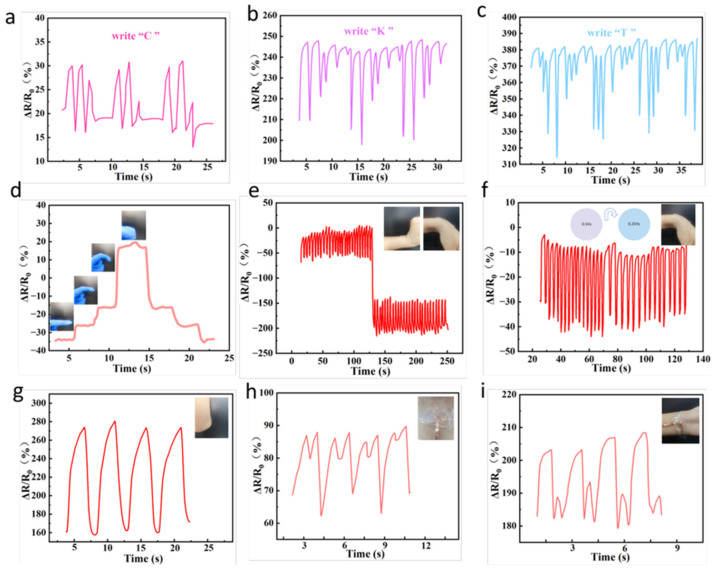
(**a**–**c**) Writing the letters “C”, “K”, and “T” on the A/G/Li^+^/Ca^2+^ hydrogel sensor. (**d**–**i**) Responsiveness of the A/G/Li^+^/Ca^2+^ hydrogel sensor to human movements: (**d**) Finger flexion, (**e**) Wrist extension and flexion, (**f**) Wrist flexion at different frequencies, (**g**) Elbow flexion, (**h**) Saying “start”, and (**i**) Pulse beat.

Leveraging the exceptional conductivity of AG/Li^+^/Ca^2+^ hydrogels, we constructed a pressure sensor and designed a sensor with writing responsiveness. By writing letters on the sensor, we recorded its resistance changes, as shown in Figure 6a–c, where the sensor sequentially wrote three distinct letters: C, K, and T. Each letter corresponded to characteristic resistance change scores and resistance values. The recorded resistance response curves for writing the same letter consistently demonstrated excellent stability and repeatability. We thoroughly evaluated the feasibility of the hydrogel strain sensor in practical applications by applying it to multiple active body regions, including fingers, wrist extension and flexion, wrist flexion at different frequencies, elbows, throats, and pulses (Figure 6d–i and Figure S5). Results demonstrate that the hydrogel-based sensors can not only sensitively monitor large-motion joint flexions like fingers, wrists, and elbows but also capture subtle changes such as throat pulses. This exhibits outstanding sensing sensitivity and stability, further validating the broad application prospects of hydrogels in flexible wearable technology.

### 3.7. AG/Li^+^/Ca^2+^ Hydrogels for Animal Monitoring

**Figure 7:**
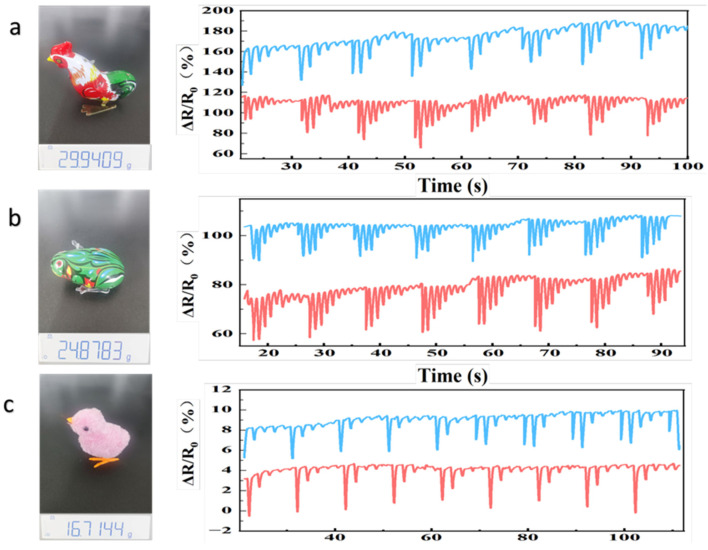
(**a**) Rooster weight and relative resistance changes during movement at −20 °C and 20 °C on A/G/Li^+^/Ca^2+^ hydrogel sensors. (**b**) Frog weight and relative resistance changes at −20 °C and 20 °C on A/G/Li^+^/Ca^2+^ hydrogel sensors. (**c**) Chick weight and relative resistance changes at −20 °C and 20 °C on A/G/Li^+^/Ca^2+^ hydrogel sensors.

Figure 7 shows the sensing performance results of the AG/Li^+^/Ca^2+^ hydrogel sensor for different simulated animal models (rooster, frog, and chick-shaped objects) at 20 °C (ambient temperature) and −20 °C (low temperature). Three representative simulated animal models with distinct weights were selected to cover the common weight range for monitoring small- to medium-sized animals: (Figure S6: the rooster-shaped object (Figure 7a)) weighed 299.409 g, the frog-shaped object (Figure 7b) weighed 248.783 g, and the chick-shaped object (Figure 7c) weighed 167.144 g. Photographs of the physical objects display synchronized electronic balance readings to ensure weight parameter accuracy. Regarding the sensing signal characteristics across models, all three simulated animal models exhibited stable ΔR/R_0_ responses at both temperatures: for the chick model in Figure 7c, ΔR/R_0_ stabilized between 10% and 15% at 20 °C, with fluctuations ranging from 11% to 16% at −20 °C (a deviation < 2%). When simulating minor movements like walking, the curve captured a distinct response peak at approximately 2 Hz with a peak value ≈ 17%, showing no signal lag or dispersion; for the frog model in Figure 7b, ΔR/R_0_ ranged from 15% to 20% at 20 °C and 16% to 21% at −20 °C, with fluctuation margins < 4% in both cases. When simulating minor displacements like frog crawling, the curve exhibited 2–3 symmetrical peaks (peak ≈ 22–23%), demonstrating high signal discernibility; the rooster model in Figure 7a, as the heaviest test subject, exhibited stable ΔR/R_0_ values of 22–27% at 20 °C and 23–28% at −20 °C. When simulating the periodic swaying motion of a pacing rooster, ΔR/R_0_ displayed regular fluctuations with a period of approximately 1.5 s and a peak ≈ 29%, fully synchronized with the movement frequency. Notably, ΔR/R_0_ exhibits a clear linear increase with model weight. This quantitative weight-to-signal correlation enables the practical application of using sensor signals to infer object mass.

In terms of temperature stability, the AG/Li^+^/Ca^2+^ hydrogel sensor demonstrated outstanding immunity to ambient temperatures: regardless of the simulated animal model weight, the average deviation of ΔR/R_0_ at −20 °C and 20 °C remained <5%, with no sudden signal drops, drift, or response failure due to low temperatures. Even under extreme cold conditions at −20 °C, the ionic conductive circuit within the hydrogel remained intact, with no significant inhibition of Li^+^ and Ca^2+^ migration. This performance stems from Ca^2+^ disrupting the hydrogen bond network of water molecules within the hydrogel, effectively suppressing ice crystal formation and preventing network structural collapse from affecting conductivity at low temperatures. Therefore, the AG/Li^+^/Ca^2+^ hydrogel sensor exhibits outstanding comprehensive performance in animal monitoring scenarios: not only can it accommodate monitoring subjects weighing between 167 and 300 g, enabling precise capture of weight and motion signals, but it also maintains stable sensing performance across a temperature range of −20 °C to 20 °C. This effectively addresses the critical issue of performance degradation in conventional hydrogel sensors under low-temperature conditions, laying a solid foundation for future applications in practical animal monitoring fields such as field ecological monitoring and livestock behavior tracking.

## 4. Conclusions

This study successfully developed a novel AG/Li^+^/Ca^2+^ ion-conductive hydrogel. Through the synergistic interaction between the acrylamide-gelatin dual network structure and lithium-calcium dual ions, it achieved a breakthrough enhancement in the material’s comprehensive performance. The hydrogel exhibited outstanding mechanical properties (1408.5% tensile strain), environmental stability (−20 °C freeze resistance and 97% water retention), and multifunctional sensing capabilities, enabling stable adhesion to nine types of substrate surfaces. This hydrogel, combining outstanding mechanical properties with conductivity, finds applications in wearable strain sensors capable of detecting human movements such as arm bending, finger waving, and swallowing. Particularly in animal monitoring, this hydrogel sensor successfully captured precise motion signals from diverse animal models (167–300 g) including chicks, frogs, and roosters across temperatures ranging from −20 °C to 20 °C. ΔR/R_0_ linearly increased with weight (167.144 g corresponding to 10–15%, 299.413 g corresponds to 22–27%). It precisely captures characteristic signal peaks from subtle animal movements like pecking and crawling. Within the −20 °C to 20 °C temperature range, the average sensor signal deviation remained <5%. The ionic conductive network remains intact at low temperatures, overcoming the cold-induced failure issues of conventional hydrogels and demonstrating excellent weight adaptability and thermal stability. Its unique ion-regulation strategy provides innovative solutions for practical applications of flexible electronic devices in ecological monitoring, livestock management, and related fields.

## Data Availability

Data will be made available on request.

## Supplementary Materials

The following supporting information can be downloaded at: https://www.mdpi.com/article/10.3390/s26072086/s1, Table S1: The content of Gelatin of AG/Li^+^/Ca^2+^ hydrogels. Table S2: The content of Li^+^ of AG/Li^+^/Ca^2+^ hydrogels. Table S3: The content of Ca^2+^ of AG/Li^+^/Ca^2+^ hydrogels. Figure S1: SEM images of the surface morphology of AG/Li^+^/Ca^2+^ hydrogel. Figure S2a: Cyclic tensile curve of hydrogel at 100–1200% strain. Figure S2b. Five cycles of hydrogel at 700% strain. Figure S3: Schematic diagram of the adhesion test device. Figure S4: Comparison of the weights of AG hydrogel, AG/Li^+^ hydrogel, and AG/Li^+^/Ca^2+^ hydrogel after being left at room temperature for approximately 10 days. Figure S5: Mechanism Diagram of Wearable Health Monitoring and Exercise Tracking. Figure S6: Small Animal Monitoring Schematic Diagram.

## Abbreviations

AG, acrylamide/gelatin; AG/Li^+^, acrylamide/gelatin/lithium chloride hydrogel; AG/Li^+^/Ca^2+^, acrylamide/gelatin/lithium chloride/calcium chloride hydrogel; AM, acrylamide; APS, ammonium persulfate; CaCl_2_, calcium chloride; FTIR, Fourier transform infrared; Gel, gelatin; GF, gauge factor; LiCl, lithium chloride; MBA, N,N′-methylenebisacrylamide; NTC, negative temperature coefficient; PAM, polyacrylamide; PDMS, polydimethylsiloxane; PTFE, polytetrafluoroethylene; SA, sodium alginate; SA/PAM, sodium alginate/polyacrylamide; SEM, scanning electron microscopy; TCR, temperature coefficient of resistance.

## References

[1] Jiang Y.N., Zhang X., Zhang W., Wang M.H., Yan L.W., Wang K.F., Han L., Lu X. (2022). Infant skin friendly adhesive hydrogel patch activated at body temperature for bioelectronics securing and diabetic wound healing. ACS Nano.

[2] Lopez C. (2023). Artificial intelligence and advanced materials. Adv. Mater..

[3] Yu X., Li H., Wang J., Zhang X., Jiao R., Ren Y., Zhang D., Ling N., Ye Y. (2025). Recent advances and future prospects of wearable sensors based on nanomaterial sensing mechanisms for biosafety monitoring. Chem. Eng. J..

[4] Gao J., Li X., Xu L., Yan M., Bi H., Wang Q. (2024). Transparent multifunctional cellulose-based conductive hydrogel for wearable strain sensors and arrays. Carbohydr. Polym..

[5] Xu J., Ma J., Peng Y., Cao S., Zhang S., Pang H. (2023). Applications of metal nanoparticles/metal-organic frameworks composites in sensing field. Chin. Chem. Lett..

[6] Suganthi S., Ahmad K., Oh T.H. (2025). Progress in graphitic carbon nitride-based composite materials for electrochemical sensing and energy storage applications. ChemistrySelect.

[7] Wang Y., Zhao Z., Wang Y., Liu Z., Chen L., Qi J., Xie Y., Zhao P., Fei J. (2025). Ultrafine metal-organic framework@graphitic carbon with MoS_2_-CNTs nanocomposites as carbon-based electrochemical sensor for ultrasensitive detection of catechin in beverages. Microchim. Acta.

[8] Zhu T., Ni Y., Biesold G.M., Cheng Y., Ge M., Li H., Huang J., Lin Z., Lai Y. (2023). Recent advances in conductive hydrogels: Classifications, properties, and applications. Chem. Soc. Rev..

[9] Wang L., Xu T., Zhang X. (2021). Multifunctional Conductive Hydrogel-Based Flexible Wearable Sensors. Trends Anal. Chem..

[10] Hui Z., Zhang Z., Wang Y., Zhang R., Liu X., Jiang M., Ju F., Hou W., Xia Z., Wang D. (2024). Gradiently foaming ultrasoft hydrogel with stop holes for highly deformable, crack-resistant and sensitive conformal human-machine interfaces. Adv. Mater..

[11] Ji Y., Li T., Abo-Dief H.M., Abualnaja K.M., Wei M., Zhang J., Wang X., Zhang J., Guo Z., El-Bahy Z.M. (2024). Polyacrylamide/starch hydrogels doped with layered double hydroxides towards strain sensing applications. Int. J. Biol. Macromol..

[12] Ge G., Lu Y., Qu X., Zhao W., Ren Y., Wang W., Wang Q., Huang W., Dong X. (2020). Muscle-Inspired Self-Healing Hydrogels for Strain and Temperature Sensor. ACS Nano.

[13] Chen J.H., Zhang L., Tu Y.Y., Zhang Q., Peng F., Zeng M., Zhang M.Q., Tao X.M. (2021). Wearable self-powered human motion sensors based on highly stretchable quasi-solid state hydrogel. Nano Energy.

[14] Kong D., El-Bahy Z.M., Algadi H., Li T., El-Bahy S.M., Nassan M.A., Li J., Faheim A.A., Li A., Xu C. (2022). Highly sensitive strain sensors with wide operation range from strong MXene-composited polyvinyl alcohol/sodium carboxymethylcellulose double network hydrogel. Adv. Compos. Hybrid Mater..

[15] Gao W., Emaminejad S., Nyein H.Y.Y., Challa S., Chen K., Peck A., Fahad H.M., Ota H., Shiraki H., Kiriya D. (2016). Fully Integrated Wearable Sensor Arrays for Multiplexed In Situ Perspiration Analysis. Nature.

[16] Wu J., Han S., Yang T., Li Z., Wu Z., Gui X., Tao K., Miao J., Norford L.K., Liu C. (2018). Highly Stretchable and Transparent Thermistor Based on Self-Healing Double Network Hydrogel. ACS Appl. Mater. Interfaces.

[17] Yao D., Wang W., Wang H., Luo Y., Ding H., Gu Y., Wu H., Tao K., Yang B.R., Pan S. (2025). Ultrasensitive and breathable hydrogel fiber-based strain sensors enabled by customized crack design for wireless sign language recognition. Adv. Funct. Mater..

[18] Han S., Tan H., Wei J., Yuan H., Li S., Yang P., Mi H., Liu C., Shen C. (2023). Surface modification of super arborized silica for flexible and wearable ultrafast-response strain sensors with low hysteresis. Adv. Sci..

[19] Li S., Liu G., Wen H., Liu G., Wang H., Ye M., Yang Y., Guo W., Liu Y. (2022). A skin-like pressure-and vibration-sensitive tactile sensor based on polyacrylamide/silk fibroin elastomer. Adv. Funct. Mater..

[20] Fu Z., Li D., Liu H., Liu R., Lyu Q., Han Y., Wang Y., Zhang K., Chen G., Tian Y. (2024). Antifreeze protein-based ultrastretchable and highly sensitive hydrogel for human-machine interaction. Chem. Eng. J..

[21] Liu R., Liu Y., Fu S., Cheng Y., Jin K., Ma J., Wan Y., Tian Y. (2024). Humidity adaptive antifreeze hydrogel sensor for intelligent control and human-computer interaction. Small.

[22] Li X., Yang H., Lu S., Cai R. (2023). A flexible strain sensor with high electrical conductivity, rapid electrothermal response, and high strain sensitivity for real-time monitoring of human joint movement. Adv. Mater. Technol..

[23] Shi Y., Guan Y., Liu M., Kang X., Tian Y., Deng W., Yu P., Ning C., Zhou L., Fu R. (2024). Tough, antifreezing, and piezoelectric organohydrogel as a flexible wearable sensor for human-machine interaction. ACS Nano.

[24] Bai Y., Zhou Y., Wu X., Yin M., Yin L., Qu S., Zhang F., Li K., Huang Y. (2025). Flexible strain sensors with ultra-high sensitivity and wide range enabled by crack-modulated electrical pathways. Nano-Micro Lett..

[25] Zhao D., Feng M., Zhang L., He B., Chen X., Sun J. (2021). Facile synthesis of self-healing and layered sodium alginate/polyacrylamide hydrogel promoted by dynamic hydrogen bond. Carbohydr. Polym..

[26] Yan X., Yang J., Chen F., Zhu L., Tang Z., Qin G., Chen Q., Chen G. (2018). Mechanical properties of gelatin/polyacrylamide/graphene oxide nanocomposite double-network hydrogels. Compos. Sci. Technol..

[27] Guo R., Bao Y., Zheng X., Zhang W., Liu C., Chen J., Xu J., Wang L., Ma J. (2023). Hofmeister effect assisted dual-dynamic-bond cross-linked organohydrogels with enhanced ionic conductivity and balanced mechanical properties for flexible sensors. Adv. Funct. Mater..

[28] Lei T., Wang Y., Feng Y., Duan X., Zhang Q., Wan A., Xia Z., Shou W., Fan J. (2025). PNIPAAm-based temperature responsive ionic conductive hydrogels for flexible strain and temperature sensing. J. Colloid Interface Sci..

[29] He F., You X., Gong H., Yang Y., Bai T., Wang W., Guo W., Liu X., Ye M. (2020). Stretchable, biocompatible, and multifunctional silk fibroin-based hydrogels toward wearable strain/pressure sensors and triboelectric nanogenerators. ACS Appl. Mater. Interfaces.

[30] Zhang J., Hu Y., Zhang L., Zhou J., Lu A. (2023). Transparent, ultrastretching, tough, adhesive carboxyethyl chitin/polyacrylamide hydrogel toward high-performance soft electronics. Nano-Micro Lett..

[31] Ma J., Liang H., Li W., Liang E., Zhang W. (2025). Polyacrylamide-Gelatin-MXene Composite Hydrogels with Interpenetrating Network Structures for Human Movement Monitoring. ACS Appl. Polym. Mater..

[32] Chen T., Chen X., Lin Z., Zhang Y., Zhao G., Xu L., Peng X., Wu B. (2026). Biodegradable, Stretchable, and Self-Healing Starch-Based Hydrogel with Intelligent Multi-Bond Network Facilitated by MXene Nanosheets for Multifunctional Wearable Electronics. Adv. Mater..

[33] Liu Y., Tian G., Du Y., Shi P., Li N., Li Y., Qin Z., Jiao T., He X. (2024). Highly stretchable, low-hysteresis, and adhesive TA@MXene-composited organohydrogels for durable wearable sensors. Adv. Funct. Mater..

[34] Zhao W., Zhou H., Li W., Chen M., Zhou M., Zhao L. (2024). An environment-tolerant ion-conducting double-network composite hydrogel for high-performance flexible electronic devices. Nano-Micro Lett..

[35] Zhao Y., Wu R., Hao Y., Zhao Y., Zhang X., Liu H., Zhai W., Dai K., Pan C., Liu C. (2025). Eco-Friendly Multifunctional Hydrogel Sensors Enabled Sustainable and Accurate Human-Machine Interaction System. Adv. Mater..

[36] Cao P., Wei J., Zhang T., Deng H., Han Y., Chen Z., Chen Y., Guo Y., Ma C. (2025). Multifunctional Luffa Sponge Hydrogel with High Mechanical Strength, Fatigue Resistance, and Ionic Conductivity for Monitoring Human Vital Signs. Adv. Funct. Mater..

[37] Cui D., Sun Y., Li T., Hu Z., Zhao Y., Wang X., Chen S., Toktarbay Z., Wei H. (2025). Mechanically robust polyacrylamide/gelatin ionic hydrogels reinforced by sodium alginate for wearable device applications. Adv. Compos. Hybrid Mater..

[38] Yan X., Chen Q., Zhu L., Chen H., Wei D., Chen F., Tang Z., Yang J., Zheng J. (2017). High strength and self-healable gelatin/polyacrylamide double network hydrogels. J. Mater. Chem. B.

[39] Zhang Y., Dai Y., Xia F., Zhang X. (2022). Gelatin/polyacrylamide ionic conductive hydrogel with skin temperature-triggered adhesion for human motion sensing and body heat harvesting. Nano Energy.

