# Supplementary material for: Low-Temperature One-Pot Fabrication of a Dual-Ion Conductive Hydrogel for Biological Monitoring
Source: Sensors (Basel). 2026 Mar 27;26(7):2086. doi: 10.3390/s26072086 (PMC13074432; doi:10.3390/s26072086)
Supplement: Supplementary file 1 [file sensors-26-02086-s001.zip › sensors-4175053-supplementary.pdf]

Supplementary Information

Supplementary Tables

Table S1. The content of Gelatin of AG/Li^+/Ca^{2+} hydrogels

Samples	Gel（g）	LiCl（g）	$CaCl_2$（g）	AM（g）	MBA（g）	APS（g）	water（g）
0.7wt%Gel	0.05	0.6	0.2	1	0.002	0.02g	7ml
1.4 wt%Gel	0.1	0.6	0.2	1	0.002	0.02g	7ml
2.1 wt%Gel	0.15	0.6	0.2	1	0.002	0.02g	7ml
2.8 wt%Gel	0.2	0.6	0.2	1	0.002	0.02g	7ml
3.5 wt%Gel	0.25	0.6	0.2	1	0.002	0.02g	7ml

Table S2. The content of Li^+ of AG/Li^+/Ca^{2+} hydrogels

Samples	Gel（g）	LiCl（g）	$CaCl_2$（g）	AM（g）	MBA（g）	APS（g）	water（g）
0.7wt% Li^+	0.15	0.3	0.2	1	0.002	0.02g	7ml
1.4 wt% Li^+	0.15	0.6	0.2	1	0.002	0.02g	7ml
2.1 wt% Li^+	0.15	0.9	0.2	1	0.002	0.02g	7ml
2.8 wt% Li^+	0.15	1.2	0.2	1	0.002	0.02g	7ml
3.5 wt% Li^+	0.15	1.5	0.2	1	0.002	0.02g	7ml

Table S3. The content of Ca^{2+} of AG/Li^+/Ca^{2+} hydrogels

Samples	Gel（g）	LiCl（g）	$CaCl_2$（g）	AM（g）	MBA（g）	APS（g）	water（g）
0.5wt% Ca^{2+}	0.15	0.6	0.1	1	0.002	0.02g	7ml
1.0 wt% Ca^{2+}	0.15	0.6	0.2	1	0.002	0.02g	7ml
1.5 wt% Ca^{2+}	0.15	0.6	0.3	1	0.002	0.02g	7ml
2.0 wt% Ca^{2+}	0.15	0.6	0.4	1	0.002	0.02g	7ml
2.5 wt% Ca^{2+}	0.15	0.6	0.5	1	0.002	0.02g	7ml

Supplementary Figures

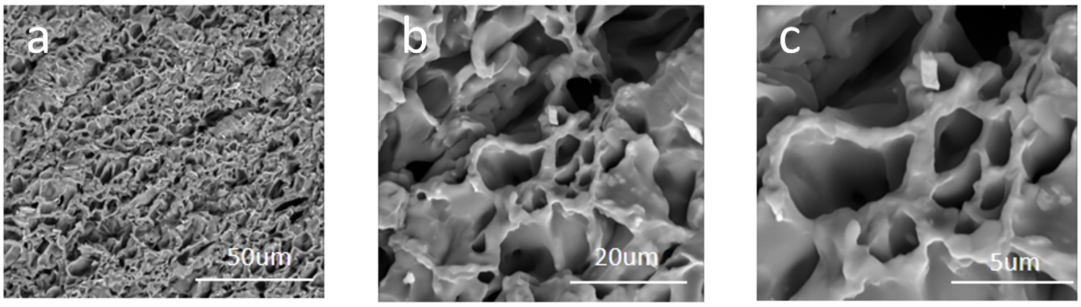

Figure S1SEM images of the surface morphology of AG/Li^{+}/Ca^{2+} hydrogel

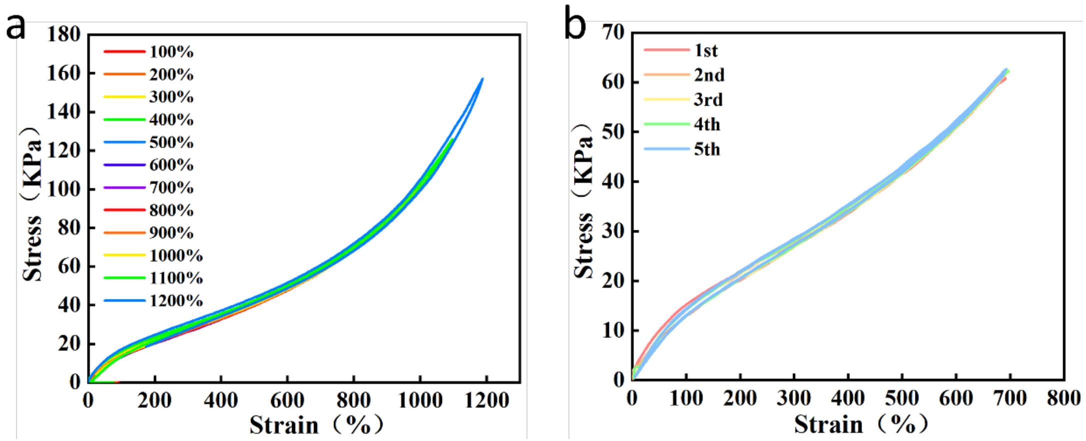

Figure S2 a. Cyclic tensile curve of hydrogel at 100%-1200% strain .b. Five cycles of hydrogel at 700% strain

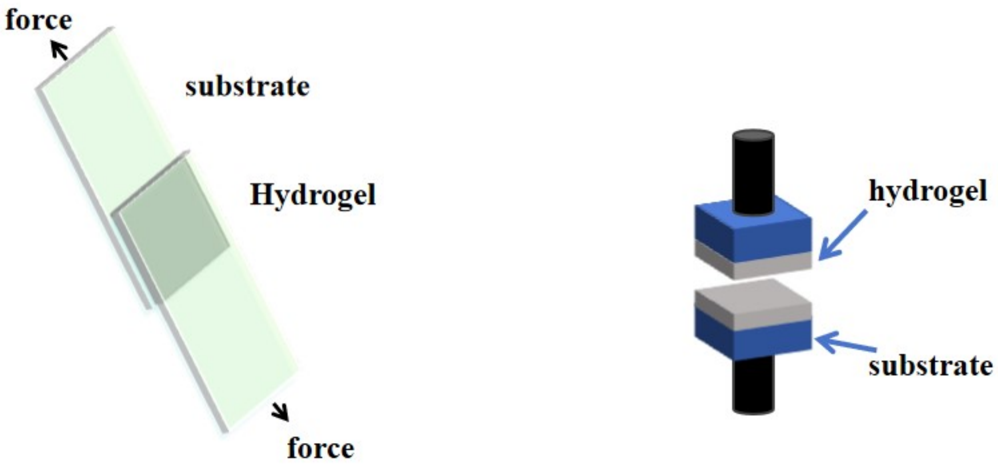

Figure S3 Schematic diagram of the adhesion test device

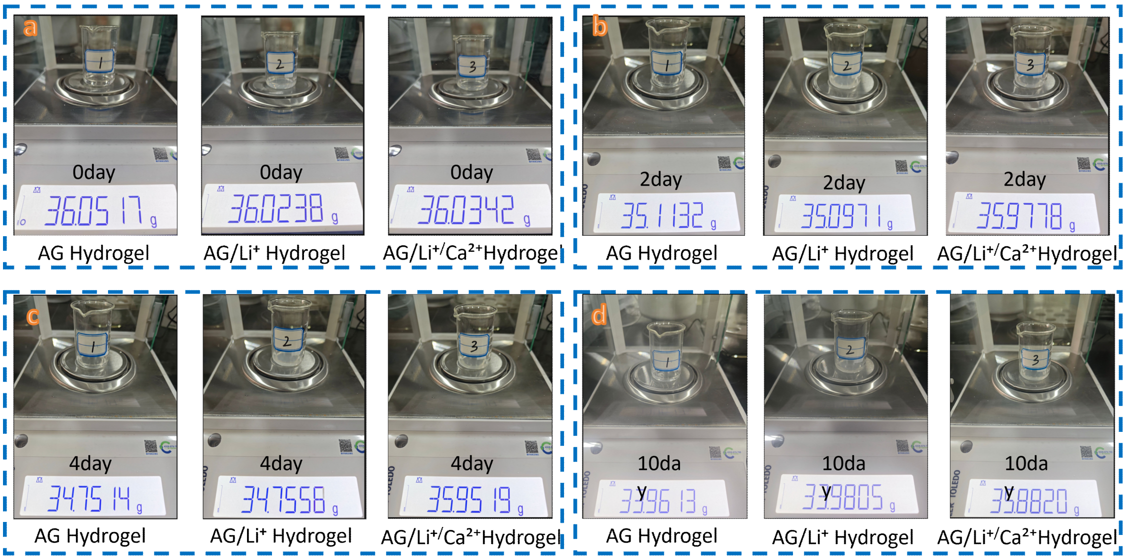

Figure S4 Comparison of the weights of AG hydrogel, AG/Li^+ hydrogel, and AG/Li^+/Ca^{2+} hydrogel after being left at room temperature for approximately 10 days

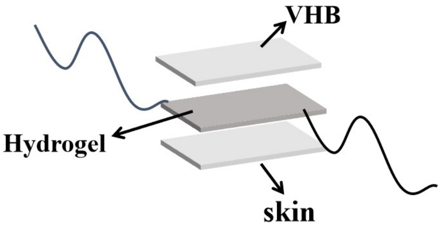

Figure S5 Mechanism Diagram of Wearable Health Monitoring and Exercise Tracking

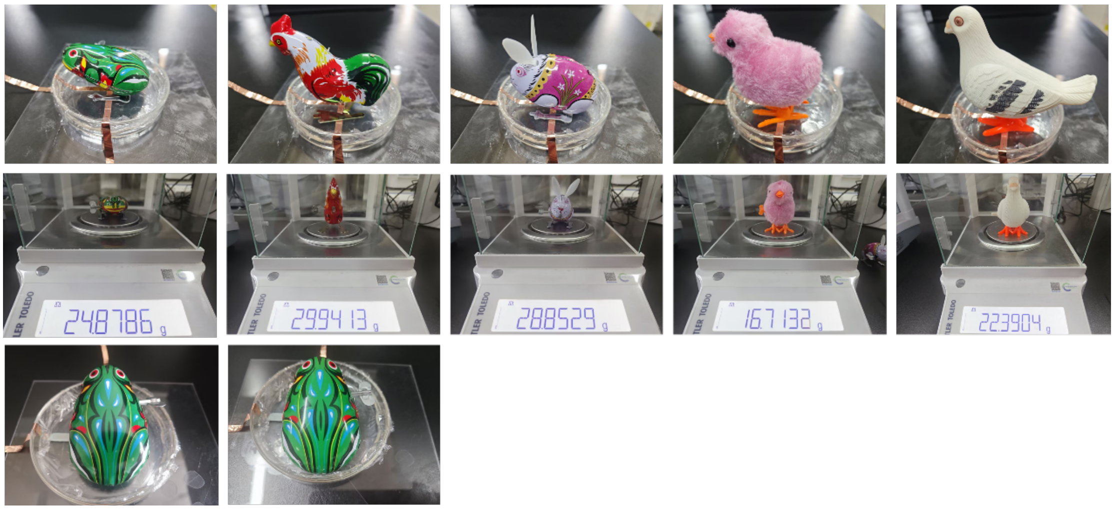

Figure S6 Small Animal Monitoring Schematic Diagram
